# A full-factorial randomized controlled trial of adjunct couples HIV testing and counseling components addressing drug use and communication skills among sexual minority male couples

**DOI:** 10.1186/s12889-021-12208-3

**Published:** 2021-11-24

**Authors:** Tyrel J. Starks, Kory D. Kyre, Christine B. Cowles, Juan Castiblanco, Catherine Washington, Jayelin N. Parker, Erin M. Kahle, Rob Stephenson

**Affiliations:** 1 Department of Psychology, Hunter College of the City University of New York (CUNY), New York, NY USA; 2 Doctoral Program in Health Psychology and Clinical Science, Graduate Center of CUNY, New York, NY USA; 3 Department of Systems, Populations and Leadership, and The Center for Sexuality and Health Disparities, School of Nursing, University of Michigan, Ann Arbor, MI USA; 4 Department of Health Behavior and Biological Sciences, and The Center for Sexuality and Health Disparities, School of Nursing, University of Michigan, Ann Arbor, MI USA

**Keywords:** Club drugs, Gay and bisexual men, Men who have sex with men, Relationships, Emerging adults

## Abstract

**Background:**

The past decade has seen increasing attention directed to the development of HIV prevention interventions for male couples, driven by epidemiological data indicating that main or primary – rather than causal – partnerships account for a substantial number of HIV infections in this population. Couples HIV testing and counseling (CHTC) has emerged as a standard of care in the US. This protocol describes a study that aims to evaluate the efficacy of two adjunct components to CHTC – communication training (CT) videos and a substance use module (SUM) – to reduce drug use and sexual HIV transmission risk behavior.

**Methods:**

Eligible couples must include one participant who is aged 17-29, HIV-negative, and reports recent drug use. Both partners must be aged 17 or older, identify as cismale (assigned male sex at birth and currently identify as male gender), and communicate in English. Couples are randomized post-baseline to one of four conditions (CHTC as usual, CHTC plus CT video; CHTC + SUM and CHTC + CT video + SUM) in a full-factorial design. Follow up assessments are completed at 3-, 6-, 9- and 12-months post baseline.

**Discussion:**

Results of this trial will enhance the application of CHTC. If found effective, adjunct components would comprise a brief and scalable drug use intervention that could be readily integrated into existing HIV testing settings.

**Trial registration:**

ClinicalTrials.gov Protocol Registration; NCT05000866; completed August 3, 2021; https://register.clinicaltrials.gov/

Protocol version 1.0; September 1, 2021.

## Introduction

### Background

For a little more than a decade, considerable attention has been focused on the sexual health needs of sexual minority men (SMM) in same-sex relationships (including gay, bisexual and other men who have sex with men). The impetus for much of this work was provided by groundbreaking research that estimated 35-68% of new HIV infections among SMM were transmitted between main – or primary – relationship partners rather than casual sex partners [2, 3, 32]. Risk for main partner HIV infection was particularly high among younger SMM, with estimates as high as 79% among those aged 18-29 years [32]. These studies suggested that main partner HIV transmission risk may arise from the fact that SMM have sex with their main partners more frequently and are less likely to use condoms when having sex with their main partner. More recently, Starks et al. [28] identified that partnered SMM are more likely to have sex with casual partners on days they also have sex with their main partners. The co-occurrence of these behaviors may also enhance shared risk for HIV.

In response to the observed need for dyadic sexual health interventions for this population, couples HIV testing and counseling (CHTC) – originally developed for heterosexual couples in African nations with generalized epidemics – was adapted for use with male couples in the United States [4, 30]. CHTC is an 8-step intervention during which couples receive an HIV test together and learn their status together. The HIV tester also engages the couple in a discussion that reviews their current HIV prevention practices, clarifies their sexual agreement – and expectations around communication if the agreement were broken – and develops a shared HIV prevention plan with the couple.

More recent research supports the need for ongoing attention to this population. Evidence suggests that single SMM and those in non-monogamous relationships (where sex with casual partners is in some way permitted) engage in condomless anal sex (CAS) with casual partners at comparable rates [22, 26]. Furthermore, there are indications that SMM in monogamous relationships (wherein partners have agreed to forego sex with partners outside their relationship) who break their agreement and engage in CAS with casual partners may actually do so more frequently than non-monogamous and single men [22].

The use of illicit drugs has been consistently linked to sexual risk taking among SMM. In particular, the use of a number of illicit drugs (i.e., cocaine or crack, methamphetamine, ecstasy, ketamine, gamma-hydroxybuterate – GHB) has been associated with either the occurrence or frequency of CAS with casual partners across a range of studies (e.g., [1, 15, 16, 22, 23]). In their recent paper, Starks et al. examined data from a sample of more than 65,000 SMM and found that this association was significant across relationship status and sexual agreements, though it was significantly stronger among single and non-monogamous men compared to those in monogamous relationships [22]. Separately, Starks et al. [28] have since observed an association between illicit drug use and CAS with casual partners in day-level data obtained from male couples.

Starks et al. [20] posited that CHTC – which in its standard form involves the negotiation of a sexual agreement and joint HIV prevention planning – might provide an opportunity for male couples to develop consensus related to rules or limitations on drug use. Furthermore, they hypothesized that augmenting CHTC with supplemental communication skills training might also enhance its efficacy. The team developed a communication training (CT) video to deliver dyadic skills training following a standard Cognitive Behavioral modeling paradigm. They also developed a substance use module (SUM) wherein partners create a calendar feedback tool, which is subsequently debriefed using a brief dose of Motivational Interviewing (MI). The MI component is delivered following the framework for couples MI derived by Starks et al. [24, 27].

Initial pilot results [20] indicated that completion of the SUM alone was associated with statistically significant decreases in the odds of drug use (*B* = − 3.62; *p* = .03) and drug use related problems as measured by the Drug Abuse Screening Test (DAST-10) [17] (*B* = − 0.75; *p* = .02) at 1-month post-intervention. While CT videos were not independently associated with reductions in drug use or DAST-10 scores; they significantly enhanced the long-term benefits of SUM completion. At 3-month and 6-month follow-up the SUM was only associated with significant decreases in the odds of drug use (*B* = − 2.79; *p* = .013 and *B* = − 3.93; *p* = .014 respectively) and DAST-10 scores (*B* = − 0.64; *p* = .01 and *B* = − 0.79; *p* = .003 respectively) among men who viewed the CT videos. In a direct comparison between the CHTC as usual condition and those participants who received both *We Test* adjunct components (CHTC+SUM+CT video), the combined condition had significantly lower odds of drug use at 1- and 3-month follow up and significantly lower DAST-10 scores at all follow-up time-points.

While promising, the results of Starks et al. [20] were limited in several ways. First, their study did not include biological markers for drug use and sexual risk taking to validate self-report responses. Second, the most distal follow-up was 6-months post-intervention. This was not sufficiently long enough to observe decay in the treatment effect for substance use outcomes. This limited the development of guidance or recommendations on the timing of CHTC-retesting with *We Test* module delivery. The next step in this program of research is to conduct a full scale efficacy trial that incorporates the use of biological markers in addition to self-reported behavioral data and a more extensive follow-up period with an expanded potential to observe decay in the intervention effect.

### Objectives

The purpose of the current study is to evaluate the efficacy of CT and SUM adjunct modules that augment the existing CHTC protocol. Our primary hypotheses are that completion of the SUM will be associated with a statistically significant decrease in drug use and drug-related problems at all follow-ups. We also hypothesize that viewing CT videos will enhance the effect of SUM. Based upon our initial results, we anticipate that there will not be a direct effect of adjunct intervention components on HIV Transmission Risk Behavior (TRB); however, we have powered the study to examine indirect effects. We therefore propose secondary hypotheses in which the SUM and CT components significantly reduce sexual HIV transmission risk behavior as a result of decreases in drug use.

## Methods

### Trial design

This study utilizes a randomized controlled trial design to evaluate the efficacy of two adjunct intervention components for couples HIV testing and counseling (CHTC). Participants are randomized in a full-factorial design to one of 4 conditions: CHTC as usual; CHTC + CT videos; CHTC + SUM; or CHTC plus both adjunct components.

### Rationale for comparison condition

The use of CHTC as usual, as opposed to a no-treatment control reflects the status of CHTC as the standard of care for HIV testing among partnered SMM [34].

### Study setting

All research staff are based in university research centers at Hunter College of the City University of New York or the University of Michigan, Ann Arbor. All study assessments and intervention sessions are conducted remotely via Health Insurance Portability and Accountability Act (HIPAA)-compliant, video conferencing software. Participants must reside in either the New York, NY or Detroit metropolitan areas and have access to a device capable of conducting remote study visits.

### Eligibility criteria

Participants must fulfill the following inclusion criteria to be enrolled in the study. In each couple, both partners must indicate cismale gender and be 17 years of age or older. In addition, at least one participant in each couple must (1) be aged 17 to 29 years; (2) self-report an HIV-negative or unknown sero-status; (3) report use of at least 1 illicit drug (cocaine/crack, opiates, misuse of prescription medication, stimulants, psychedelics, ecstasy, ketamine, GHB) in the past 30 days, (4) have engaged in CAS with a casual partner or a main partner who is non-monogamous or sero-discordant in the past 90 days, (5) live in the New York City or Detroit metropolitan area, and (6) be able to speak and read in English.

Participants will be excluded from the study if they indicate signs of acute suicidality; gross impairment in cognitive functioning; or any history of intimate partner violence and current safety concerns in the current relationship. The trial does not place restrictions on concomitant receipt of care for substance use or HIV prevention.

### Interventions

All participants, regardless of condition, will complete a CHTC session. Completion of CHTC involves 8-steps: 1.) Introduce CHTC and Obtain Concurrence: an introduction to the CHTC process and receiving the couples consent; 2.) Prepare For and Conduct HIV Test: explanation of the HIV test itself and possible results couples could receive, followed by a rapid HIV test; 3.) Explore Couple’s Relationship: an exploration of the couple’s relationship to build rapport; 4.) Discuss HIV Risk Concerns and Reasons for Seeking CHTC: discussion of current risks, reasons for testing and HIV prevention strategies (i.e. condom use, PrEP use, etc.); 5.) Discuss Couple’s Agreement: exploration of the couple’s sexual agreement and rules in regards to outside sexual partners; 6.) Provide Results: results are given to each member of the couple at the same time and can be the same (concordant negative or positive) or discordant; 7.) Develop Care, Treatment, and Prevention Plan Based on Result: discussion of future steps regarding HIV transmission risk prevention strategies (i.e. discussion of introducing condoms or PrEP use); and 8.) Link with Follow-up Services: referrals given in light of current HIV test results (i.e. referrals for confirmatory testing and other health services).

### Communication training (CT) videos

The CT video component is designed to be self-delivered. Four couples are depicted in separate scenes discussing HIV testing, drug use, sexual agreements, and drug use during sex. Each scene is viewed twice. In the initial viewing, the couple in the video makes one or more communication errors. The scene is subsequently viewed a second time and the couple utilizes more effective communication skills, resulting in a more adaptive resolution. Each scene is introduced by a narrator who points out communication errors and orients viewers to skills utilized in adaptive versions of each scene.

### Substance use module (SUM)

The SUM is administered after step 5 of CHTC and prior to the delivery of rapid HIV-test results. The couple is first asked to fill in a calendar for the past 30 days, indicating each day on which either member used drugs or consumed alcohol. Completion of the calendar is done using an excel spreadsheet displayed on a shared computer screen to facilitate remote intervention delivery. After completion of the calendar, the HIV tester asks a series of debriefing questions designed to elicit the couples’ perspective on their use, establish the couple’s goals and limits for drug use, and make plans to achieve these goals.

### Intervention training

All CHTC interventionists and supervisors – regardless of site – complete a training sequence that includes CHTC and MI components. MI training involves attendance at a 2-day workshop that covers basic MI concepts, processes, and skills as well as strategies, processes and techniques unique to the delivery of MI with couples. CHTC training follows Sullivan and Stephenson’s curriculum, as adopted by CDC. Afterwards, providers complete a series of mock sessions. These begin with individual MI mocks to consolidate basic counseling skills. Subsequently a sequence of 4 CHTC mocks is completed so providers can practice each formulation of HIV test result delivery (concordant negative, concordant positive, and serodiscordant). The last 3 CHTC mocks include the SUM component.

### Fidelity monitoring and supervision

Drs. Starks or Stephenson provide feedback on all mocks based upon review of audio recordings. In addition, mock sessions are recorded and fidelity assessed using the CDC’s CHTC fidelity checklist [33]. Fidelity to MI during SUM completion is evaluated using a modified version of the Motivational Interviewing Treatment Integrity system Moyers et al. [11] augmented with codes developed by Starks et al. [21] specifically to assess the fidelity of MI delivered to couples.

### Primary outcomes

#### Illicit drug use

Will be operationalized as the odds of drug use in the past 30 days indicated by self-report on the Time-line Follow-back (TLFB) interview. Biologically, the occurrence of drug use will be assessed via urine assay.

#### HIV transmission risk behavior (TRB)

Primary Analyses focus on three dichotomous behavioral indicators of TRB These include: (1) Condomless anal sex (CAS) with a casual partner in the absence of PrEP; (2) CAS with a sero-discordant main partner (whose viral load is detectible) or concurrent CAS between main and casual partners in the absence of PrEP; (3) Any chlamydia or gonorrhea diagnosis in the absence of PrEP.

### Participant timeline

Figure 1

**Fig. 1:**
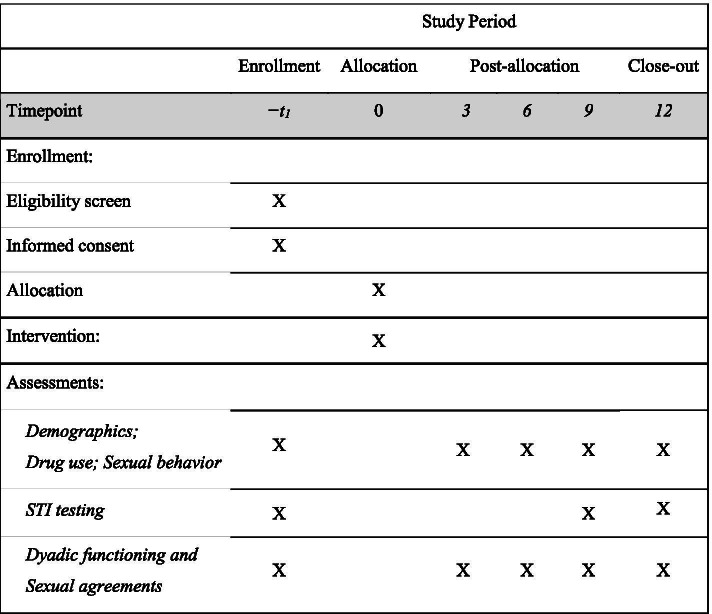
Participant Timeline

#### Sample size

Power analyses were generated by Monte Carlo simulation using Mplus (version 8.0). All estimates were based upon 10,000 sample replications and power was defined as the percentage of randomly generated samples in which the specified parameter was statistically significant. All simulated models were multi-level, nesting participants (at Level 1) within couples (at Level 2). Models focused on detecting between-condition differences at any one follow-up time-point (as opposed to predicting slope factors). This allowed for more direct extrapolation of error variances from existing baseline data and is consistent with the primary aim of the study. The main effects of the two interventions (the drug use calendar and video-based communication skills training) as well as their interaction were modeled at level 2. Based upon the results of our previous studies, we anticipate 80% minimum follow-up retention at 12 months. These analyses therefore reflect a conservative analytic sample of 240 couples (480 individuals).

Analyses indicated that the proposed study has power > .80 to detect a simple main effect of SUM and/or CT videos corresponding to a 15% reduction in the odds of drug use and a 1.0 point reduction in DAST scores at any follow-up time point. The proposed study has power > .80 to detect a 2% reduction in the odds of reporting HIV TRB. Effects of these magnitudes are plausible given our pilot data. *We Test* pilot data have indicated that mediational pathways linking CT videos with drug use outcomes likely involve depression. The effects of viewing ACTV were generally small across indices of communication skills and psychological functioning. The most promising (statistically significant) effects were associated with improvements in self- disclosure communication (β = .13) and depression (β = −.16). Notably, the indirect pathway between CT and depression through self-disclosure communication was of moderate size (β = −.12) indicating that communication skills may be one mechanism by which ACTV yields reductions in depression. Depression scores were, in turn, positively associated with the odds of drug use (β = −.45) and DAST scores (β = .06). Using these effects as preliminary estimates, Monte Carlo simulations suggested that the proposed study design has power > .80 to detect the significance of direct pathways and implied indirect effects of intervention.

#### Recruitment

We will utilize a multifaceted recruitment effort including both active and passive approaches. Outreach will be primarily conducted through advertisements on popular social media and dating apps. Potential participants will be able to complete the eligibility screener directly from these advertisements and, if eligible, will be contacted by study staff to begin the process of enrollment into the study. Other avenues of recruitment, such as organized outreach through community-based organizations as well as bars and community events will also be utilized.

Prescreened eligible participants obtained through the online screener will be automatically emailed a screening link to be sent to their main partner. If eligible, participants will be contacted by study staff via phone calls, texts, and emails to schedule a remote visit to determine final eligibility and participants will review and complete the consent document. Both will then have their baseline appointment scheduled and have their home-based surveys emailed to them. Once their home-based surveys are completed, Molecular Testing Labs (MTL) STI/drug and HIV testing kits will be mailed to the participants’ home(s).

### Methods: assignment of interventions

#### Randomization

Couples will be randomly assigned to 1 of 4 conditions, CHTC as usual, CHTC + SUM, CHTC + CT video, or CHTC + SUM + CT video using a stratified block randomization procedure programmed in Qualtrics. Specifically, randomization will account for baseline report of: (1) relationship length (4 years or less/greater than 4 years) and (2) race/ethnicity makeup of the couple, for example, both partners identify as white and non-Hispanic/one or both partners identify as non-white or Hispanic. The random assignment will be performed by the assigned study interventionist immediately prior to intervention delivery.

#### Blinding

Study staff delivering intervention cannot be blinded to the condition they are delivering. Assessment staff are blinded to the condition at baseline and follow-up. There are no anticipated circumstances that would result in unblinding of assessors during the trial. Participants cannot be blinded to their assigned arm. The arms are described in the informed consent process and it is reasonable that participants will be able to deduce whether or not they viewed the CT videos and discussed drug use during their session.

### Methods: data collection, management, and analyses

#### Data collection for primary outcomes

Self-report data for all primary outcomes will be gathered using a Research Assistant administered timeline follow-back (TLFB) interview [18] covering the past 30 days. The interview administration is facilitated by the use of a calendar data entry platform supported in Microsoft Access. The Research Assistant first identifies and records “anchor dates” or significant events on the calendar. Next, for participants who are prescribed PrEP, the Research Assistant records all days the participant indicated a missed dose of PrEP. Next, the Research Assistant records days on which the participant reported heavy drinking or the use of marijuana or other illicit drugs (i.e., cocaine or crack, MDMA, methamphetamine, ketamine, or GHB). Finally, the Research Assistant records sexual events. These entries included partner type (main or casual), the sex act performed (e.g., anal insertive, anal receptive), and whether a condom was used.

Biological testing is coordinated through Molecular Testing Laboratory (MTL). All specimens are self-collected at home using materials supplied directly by MTL. MTL also provides materials necessary for return shipping. Participants complete specimen collection during their remote baseline appointment so a Research Assistant is available to clarify specimen collection procedures, answer questions as they arise, and observe the preparation of specimens for shipment.

Urine drug testing will be completed using the urethral drug testing kit by Molecular Testing Labs. Participants are provided with a collection cup and are instructed on the process of correctly collecting a urine sample by trained study staff. The participant will use a provided pipette to transfer the urine into a collection tube that will be sent back to the lab for testing. This sample will be tested for the recent use of illicit drugs and results are available within 5-7 business days.

Tests for urethral STIs will be conducted on self-collected urine specimens, which will be packaged according to appropriate biosafety protocols for return mailing to the laboratory. Tests for rectal STIs will be conducted on a self-administered rectal swab, using a Q-Tip-like swab and with written and cartoon instructions that have been used to support over 5000 rectal swab collections by Sullivan’s team. The presence of *C. trachomatis* (CT) and N. gonorrhea (NG) in self-collected urethral and rectal specimens will determined by using the Abbott Real Time CT/NG assay, an FDA-cleared real-time polymerase chain reaction (PCR) assay for direct, qualitative detection of a region of the cryptic plasmid DNA of CT and the Opa gene of NG, as previously reported [5].

Syphilis testing will be conducted on Dried Blood Spots (DBS). MTL provides a lancet for finger stick blood draw and DBS card for specimen collection. Syphilis specimens are tested with an RPR test. Initially reactive results are reported as a positive screening test, with referral for providers. If enough plasma is not available for a “neat” initial RPR, a 1:4 dilution RPR will be conducted. If negative, the negative report will be reported with a note that it was on a 1:4 dilution. This procedure lowers the sensitivity of the RPR but would still be expected to perform well for primary and secondary syphilis infections, with the exception of very early infection.

HIV testing will be conducted with all participants who self-report an HIV-negative or unknown HIV status at baseline in the context of CHTC regardless of intervention condition. Testing will be performed using the Oraquick 4th generation testing kit. During the intervention session, the participants are guided through the process of completing the test by the interventionist. Participants will self-administer the test by rubbing the testing strip against the gum-line and allowing the test to rest in the provided developing liquid for a rapid result. Results are available in 20 min and delivered to participants immediately. At follow-up, HIV-testing will be conducted individually, allowing participants the option of an Oraquick HIV test or a self-administered dried blood-spot (DBS) test to complete their HIV test.

#### Retention plan

To maximize retention, assessments are conducted individually. This reduces the burden of coordinating scheduling with relationship partners and permits the retention of participants even if relationships dissolve during the follow-up period. In addition, study staff remain in regular (at least quarterly) contact with participants through email, text messaging, and telephone (based on participant preference). This provides the opportunity to regularly update contact information and maintain consistent engagement throughout the follow-up period.

#### Data management

All survey instruments are administered using a Qualtrics-based computer-assisted survey instrument (CASI) interface. To reduce the time required to attend the remote baseline appointment, participants have the option of completing the baseline survey before the appointment . TLFB data are gathered by a trained interviewer using a data-entry system programmed in Microsoft Access (see below for additional details). The following sections also provide specific details related to the handling of biological specimens and related results. Study procedures have been reviewed by  the CUNY University Integrated Institutional Review Board (CUNY-UI IRB). In addition, we have set up a Data Safety Monitoring Board consisting of leading experts in sexual minority men with particular specific expertise in randomized controlled trials, epidemiology, statistical analysis, and clinical research broadly.

### Data analysis plan

#### Data screening procedures and analyses of attrition

We will follow standard procedures in cleaning data and examining distributional properties (means, standard deviations, skew, kurtosis, and visual representations). Subsequently, we will conduct an analysis of attrition to determine if dropout is associated with 1) demographic variables and/or 2) drug use or TRB outcomes assessed at baseline. Factors which are observed to covary significantly with attrition will be incorporated in outcome analyses. Kahle (Co-I) is an accomplished epidemiologist who is familiar with modeling procedures applicable to the modeling of variables with non-normal distributions (i.e., categorical and count variables), and we will utilize appropriate tests of association and linking functions in statistical models. Kahle will utilize Mplus (version 8.0) [12] in conducting these analyses.

Analyses will utilize a true intent-to-treat procedure involving data from all randomized couples. Mplus has a variety of options for handling partial attrition including full-information maximum likelihood estimation. Non-random and consequential missingness can also be modeled directly through the addition of latent variables which account for the probability of missingness at any time point.

#### Primary analyses of intervention effects

Outcome variables will be tested by applying a multilevel latent growth model to follow-up data, accounting for nesting within couples. Models will utilize 5 data points (Baseline, 3-, 6-, 9-, and 12-month follow up) and full-information maximum likelihood estimation to ensure that all randomized participants are included in analyses – consistent with the ITT paradigm. Models will include an intercept (representing the initial outcome value) and two slope factors. The first slope-coefficient will quantify change from baseline to post-intervention. The second will capture change over time post intervention. Mplus accommodates count and dichotomous outcomes. Growth factors will then be regressed on intervention condition at the couple level (Level II) and the effect of the intervention will be evaluated by examining the regression coefficient (and associated *p* value) associated with intervention condition for each of these factors.

#### Moderation/mediation analyses

We will conduct analyses to identify putative moderators and mediators of intervention effects. Moderation analyses will focus on demographic factors that have been linked to HIV disparities. Specifically, we will examine whether the effect of the intervention is constant across race and ethnicity, age, and indicators of socioeconomic status (income and education) in all proposed analyses. We will also examine whether intervention effects vary across couples’ HIV status, incorporating attention to viral suppression and PrEP uptake.

We utilize the actor–partner interdependence model (APIM) Kenny et al. [6] to organize individual-level mediators. APIMs are multilevel models. At the individual level (Level 1), there are two types of effects: *actor* (the association between an individual’s score on an outcome and his own score on a predictor) and *partner* (the association between an individual’s score on an outcome and his partner’s score on a predictor). Couple-level (Level 2) effects are calculated for variables where both partners share a score (e.g., relationship length).

A series of APIMs will be calculated to examine putative mediators. Due to model complexity, each drug and TRB outcome will be examined separately. As with primary outcome analyses described above, we will utilize growth modeling procedures to calculate an intercept and slope for each outcome over time. Similarly, models will also specify growth factors for mediators at Level 1 and Level 2 during the follow-up period. In this manner, growth factors for the outcome can be regressed on growth factors for the putative mediator at both Level 1 and Level 2. Subsequently, the effect of intervention (a couple-level predictor) will be determined by examining the regression coefficients associated with intervention in the prediction of growth factors for both the outcome of interest and the mediator at Level 2. Where indicated by the pattern of significant direct effects, indirect pathways from intervention condition through actor and partner effects of the mediator will be tested using bootstrapping tests of mediation. Where outcome distributions prevent the use of bootstrapping, we will utilize a model constraint approach to evaluating the significance of indirect effects.

Follow-up exploratory analysis will investigate a range of potential mediators and moderators. In particular, we will test whether drug use mediates intervention effects on sexual risk taking outcomes. In addition, individual variables assessing mental health, discrimination, perceptions of relationship functioning, and communication skills will be tested as potential mediators of intervention condition effects. Meanwhile, baseline differences in agreements, agreement satisfaction, relationship satisfaction, commitment, and decision-making power will be tested as potential moderators of intervention effects.

#### Data monitoring

This study protocol was approved by the City University of New York’s Human Research Protection Program (HRPP; Protocol Number 2020-0452) and is registered with Clinicaltrials.gov (NCT# 05000866).

A Data Safety and Monitoring Board (DSMB), comprised of three independent experts in the field of HIV prevention, substance use intervention, and biostatistics, has been convened in accordance with NIH policy. They reviewed and approved the study procedures for trial monitoring on March 19, 2021. The DSMB will convene at least annually and also in response to the occurrence of any serious adverse events.

Data on anticipated adverse events including HIV incidence and Intimate Partner Violence will be gathered in quarterly assessments and reported to the DSMB annually by the Primary Investigator. Unanticipated events and events reported spontaneously will be reported to the DSMB on an ongoing basis by the Primary Investigator and also summarized in their annual meeting report.

#### Trial modification and discontinuation

Substantive modifications to trial procedures require prior approval of the study sponsor and DSMB. In the event of such modifications, amendments would be filed with the institutional review board and the clinicaltrials.gov record would be updated. Procedures for intervention allocation will be discontinued in response to adverse event reporting under the guidance of the DSMB. There are no a priori plans to conduct interim analyses.

#### Consent

Participants will be consented verbally by a research assistant and provided consent information in writing. A waiver of documentation of consent was obtained to reduce participant burden in this remote intervention study.

#### Confidentiality

All data will be identified by a unique study ID number. All materials will be stored in databases that are HIPAA-compliant. Risk of breach of confidentiality will be minimized in several ways. Only limited study staff at each site will have access to a file linking the ID number to participants’ names; this file and participant-identifying information will be kept in a locked cabinet and in password-protected computers. Research staff will make follow-up contacts under explicit guidelines to preserve confidentiality when telephoning or mailing information to participants. All study-affiliated staff will have completed mandatory training in good clinical practice and the responsible conduct of research. Finally, a Federal Certificate of Confidentiality is granted by NIH prior to enrollment of participants.

#### Post-trial care

Participants will be provided resources to identify local referrals for ongoing HIV testing, substance use and mental health services. Participants who exhibit signs of serious mental illness or clinically significant distress in interactions with study staff will be evaluated by a study team member with training in mental health counseling and crisis risk assessment.

#### Dissemination

Sharing of data generated by this project is an essential part of our proposed activities and will be carried out in several different ways. Our team is committed to collaboration with researchers, the health and social services community, and other entities for rapid dissemination of data to inform future research and practice. We will make our results available to the community of researchers interested in the development of interventions targeting substance use, HIV prevention, and PrEP dissemination to avoid unintentional duplication of research. In addition, dissemination efforts will focus on reaching out to the health and social services community, including providers of mental health care, substance use intervention, PrEP services, and HIV-testing as well as public health and community based organizations.

Authorship in publications will be based upon intellectual contribution and guided by American Psychological Association guidelines for authorship. The study protocol will be made available on clinicaltrials.gov. There are no plans to make participant-level data available to the public.

## Discussion

CHTC uptake has been modest in the U.S. [29] despite high acceptability among SMM [14, 31]. Given the salience of main partner transmission for the U.S. HIV epidemic, expanding the reach of couples-based prevention is essential. Stephenson et al. [29] suggested this may be achieved in part through better service integration and by expanding the scope of CHTC to meet the unique antecedents of HIV experienced by male couples (i.e. substance use). Directly aligned with this, the adjunct components tested here integrate drug use intervention into CHTC. They may expand CHTC dissemination by drawing upon resources that support substance use treatment in addition to HIV testing. It may also enhance the appeal of CHTC among substance use treatment providers who might not otherwise offer the service.

This study also furthers research on applications of Motivational Interviewing with couples. Previous research on the involvement of relationship partners in Motivational Interviews have met with mixed results [7–10, 13]. Unlike these previous studies, which conceptualized couples Motivational Interviewing in terms of relationship partners involved as adjunct participants in the treatment of an identified client, the framework for couples Motivational Interviewing that underlies the SUM component tested here conceptualizes the couple in toto as the client [24, 27]. While the approach has demonstrated promise in two pilot studies [19, 20], this is the first full-scale efficacy trial of an intervention guided by this framework.

Findings from this study will be subject to a number of limitations. First, CHTC has been acknowledged as a standard of care and its efficacy has been established in previous studies. It is therefore of limited scientific utility to include a no-treatment or attention control which would allow us to quantify the effect of CHTC delivered alone. The purpose of this study is to test the efficacy of adjunct *We Test* components. We therefore forgo the ability to provide a replication of the effect size for CHTC alone. Dyadic participation may present challenges– particularly for couples with poorer dyadic functioning [25]. Results may not generalize to couples who are unable or unwilling to participate together. By selecting sites which represent urban centers of the US HIV epidemic, we maximize the feasibility and racial/ethnic diversity of our sample; however, our sample will under-represent men from rural areas. Eligibility criteria (one partner must be 17 – 29) limit life-span representation limits generalizability; however, this criterion is driven by the heightened risk and developmental salience of emerging adulthood. Exclusion of transgender people further limits generalizability. The decision reflects the limitations of our pilot data – which included only cis-male participants. It also reflects the content of CT videos which include only cis-male actors.

Despite these limitations, this study has the potential to contribute new knowledge about dyadic functioning and health among partnered SMM. This emphasis on relationship factors and sexual health reflects more than a decade of accumulating evidence that attention to main partnerships is an essential component of a comprehensive HIV prevention strategy in the US. If found efficacious, the adjunct intervention components tested here may expand the reach and application of CHTC to the treatment of substance use.

## Data Availability

Please contact the first (corresponding) author for details.

## Abbreviations

SMM: Sexual Minority Men
CAS: Condomless Anal Sex
CHTC: Couples HIV Testing and Counseling
CT Videos: Communication Training Videos
DSMB: Data Safety and Monitoring Board
GHB: Gamma-hydroxybuterate
IRB: Institutional Review Board
HIPAA: Health Insurance Portability and Accountability Act
HIV: Human Immunodeficiency Virus
MI: Motivational Interviewing
NIH: National Institutes of Health
PrEP: Pre-exposure prophylaxis
SUM: Substance Use Module
TRB: Transmission Risk Behavior
